# Exome sequencing covers >98% of mutations identified on targeted next generation sequencing panels

**DOI:** 10.1371/journal.pone.0170843

**Published:** 2017-02-02

**Authors:** Holly LaDuca, Kelly D. Farwell, Huy Vuong, Hsiao-Mei Lu, Wenbo Mu, Layla Shahmirzadi, Sha Tang, Jefferey Chen, Shruti Bhide, Elizabeth C. Chao

**Affiliations:** 1 Department of Clinical Diagnostics, Ambry Genetics, Aliso Viejo, California, United States of America; 2 Department of Pediatrics, Division of Genetics and Genomic Medicine, University of California Irvine, Irvine, California, United States of America; Tel Aviv University, ISRAEL

## Abstract

**Background:**

With the expanded availability of next generation sequencing (NGS)-based clinical genetic tests, clinicians seeking to test patients with Mendelian diseases must weigh the superior coverage of targeted gene panels with the greater number of genes included in whole exome sequencing (WES) when considering their first-tier testing approach. Here, we use an *in silico* analysis to predict the analytic sensitivity of WES using pathogenic variants identified on targeted NGS panels as a reference.

**Methods:**

Corresponding nucleotide positions for 1533 different alterations classified as pathogenic or likely pathogenic identified on targeted NGS multi-gene panel tests in our laboratory were interrogated in data from 100 randomly-selected clinical WES samples to quantify the sequence coverage at each position. Pathogenic variants represented 91 genes implicated in hereditary cancer, X-linked intellectual disability, primary ciliary dyskinesia, Marfan syndrome/aortic aneurysms, cardiomyopathies and arrhythmias.

**Results:**

When assessing coverage among 100 individual WES samples for each pathogenic variant (153,300 individual assessments), 99.7% (n = 152,798) would likely have been detected on WES. All pathogenic variants had at least some coverage on exome sequencing, with a total of 97.3% (n = 1491) detectable across all 100 individuals. For the remaining 42 pathogenic variants, the number of WES samples with adequate coverage ranged from 35 to 99. Factors such as location in GC-rich, repetitive, or homologous regions likely explain why some of these alterations were not detected across all samples. To validate study findings, a similar analysis was performed against coverage data from 60,706 exomes available through the Exome Aggregation Consortium (ExAC). Results from this validation confirmed that 98.6% (91,743,296/93,062,298) of pathogenic variants demonstrated adequate depth for detection.

**Conclusions:**

Results from this *in silico* analysis suggest that exome sequencing may achieve a diagnostic yield similar to panel-based testing for Mendelian diseases.

## Introduction

With the expanded availability of next generation sequencing (NGS)-based clinical genetic tests, clinicians are faced with the decision to pursue targeted gene panels versus whole exome sequencing (WES) as their first-tier testing approach [1]. This decision may be particularly challenging for diseases with significant genetic and phenotypic heterogeneity. It is important to consider the benefits and limitations of each approach when deciding on the best testing strategy.

Coverage is superior on NGS panels compared to WES when the amount of sequenced nucleotides is the same. In most cases, sequence-specific enrichment is performed prior to NGS to help achieve high coverage and reduce or eliminate low-coverage regions (typically ~10-50X). Commonly, Sanger sequencing is applied to regions which are recalcitrant to NGS enquiry, due to technical or biological limitations, including GC-rich regions and regions of high homology. In contrast, current estimates of coverage achieved from whole exome capture and sequencing are 90–95% at >20X, with factors such as target enrichment design, off-target capture, repetitive and GC- or AT-rich regions, copy-number variations, and structural variations posing challenges to complete capture [2–5]. Unlike NGS panels, the addition of Sanger sequencing for regions with inadequate coverage would not be time or cost-effective for WES, where more than 20,000 genes are being analyzed.

In contrast, one key drawback of targeted NGS panels is that they may become outdated rather quickly. During the time a panel is developed and validated for clinical utilization, new studies are already published identifying newly characterized disease genes. In fact, among positive WES findings, 23% are within genes characterized within the last two years and 7% are novel gene discoveries [6]. As such, an advantage of WES is the ability to sequence the entire exome at once, allowing for the analysis and interpretation of all alterations in both well characterized and novel genes, and also allowing for re-interpretation as new genetic associations are established. Additional advantages of an exome sequencing approach include the ability to analyze a significantly larger number of genes at a reasonable cost, the potential to identify novel genes, and the ability to sequence the exomes of multiple family members simultaneously in labs that offer sequencing of trios.

Early reports comparing targeted NGS panels to WES focused on exon or gene level coverage, as related to a specific gene or set of diagnostic genes [7]. Here, we aim to calculate the analytic sensitivity of WES for pathogenic variants identified on targeted NGS panels.

## Materials and methods

The internal database at Ambry Genetics (Aliso Viejo, CA) was queried for all alterations classified as pathogenic and likely pathogenic (herein referred to collectively as ‘pathogenic variants’) detected on germline targeted NGS multi-gene panel testing of over 50,000 patients in the clinical diagnostic laboratory from April 2010 until July 2014. Variants underwent thorough assessment and review of available evidence and were classified using a five-tiered classification algorithm [8] based on guidelines from the American College of Genetics and Genomics and the International Agency for Research on Cancer [9, 10]. Pathogenic variants in this study included single nucleotide substitutions and small insertion and deletion events up to 40 basepairs. Gross rearrangements, large insertions, and large deletions detected on array or multiplex ligation-dependent probe amplification-based assays were excluded from analysis. Multi-gene panels targeted a range of hereditary (Mendelian) disorders including cancer susceptibility, X-linked intellectual disability (XLID), primary ciliary dyskinesia (PCD), Marfan syndrome, thoracic aortic aneurysms and dissections, and related disorders (Marfan/TAAD), and other cardiovascular diseases such as cardiomyopathies and arrhythmias, and were selected by the ordering clinician based on the patients’ clinical history. Corresponding nucleotide positions for these pathogenic variants were interrogated against data from 100 randomly-selected patients whose samples were submitted to Ambry Genetics for clinical WES, and the coverage at each position was assessed. Pathogenic variants were interpreted as having adequate depth for detection if coverage at the respective nucleotide position on WES was ≥10X. Coverage at the first and last nucleotides was averaged for insertions, and for deletions and indels coverage was assessed for the first and last nucleotides, with the lower of the two values being used for analysis. Since all data was accessed anonymously, this research was determined to be exempt from review based on 45.CFR46.101 and is in compliance with the Helsinki Declaration (Solutions Institutional Review Board, Reference Number 1OCT14-93).

### NGS panel sequencing

Library preparation, sequencing, bioinformatics, and data analysis were performed as previously described [8, 11]. Briefly, samples were enriched for sequence targets using Raindance Thunderstorm technology (RainDance Technologies, Billerica, MA), and sequenced using paired-end, 100-cycle chemistry on the Illumina HiSeq 2000 (Illumina, San Diego, CA). Importantly, PCR duplicated sequences were removed from the dataset prior to alignment, and quantification of basepair level coverage. The sequence data were aligned to the reference human genome (GRCh37) and variant calls were generated using CASAVA and Pindel [12]. No/low-coverage regions (i.e. <50X) and variant calls other than known non-pathogenic alterations were analyzed using automated fluorescence dideoxy sequencing.

### Whole exome sequencing

Exome library preparation, sequencing, bioinformatics, and data analysis were performed as previously described [6, 13, 14]. Briefly, samples were prepared using SeqCap EZ VCRome 2.0 (Roche NimbleGen, Madison, WI) and sequenced using paired-end, 100-cycle chemistry on the Illumina HiSeq 2000 (Illumina, San Diego, CA). The sequence data were aligned to the reference human genome (GRCh37) and variant calls were generated using GATK and CASAVA. Variant filtering on WES was performed using a custom bioinformatics pipeline as previously described in detail [6]. Briefly, stepwise filtering included the removal of variants with quality scores <20 and allele counts <10X, common SNPs, intergenic and 3’/5’ UTR variants, non-splice-related intronic variants, and synonymous variants. Variants were filtered further based on family history and possible inheritance models. Data were annotated with the Ambry Variant Analyzer tool (AVA) [8]. All samples were required to meet minimum quality standards, with at least 90% of bases covered at ≥10X and having base call quality scores ≥Q20, which translates to a base-calling error rate of 1:100. Identified candidate alterations were confirmed using automated fluorescence dideoxy sequencing. All gene/bases covered in gene panel design were covered at >20X in the exome design.

## Results and discussion

A total of 1533 different pathogenic variants identified on targeted NGS multi-gene panel testing were included in this analysis, representing 91 genes implicated in 5 disease categories identified through analysis of greater than ~100,000 alleles (S1 Table). Pathogenic variants in cancer susceptibility genes accounted for 88.1% of pathogenic variants analyzed (n = 1350), with each of the other disease categories accounting for <4% of pathogenic variants studied. Exonic single nucleotide substitutions were the most common type of variant included in this analysis (n = 665, 43.4%), followed by small deletions (n = 485, 31.6%), intronic variants (n = 184, 12.0%), small duplications and insertions (n = 169, 11.0%), and indels (n = 30, 2.0%).

Considering that coverage was assessed among 100 individual WES samples for each pathogenic variant (153,300 individual assessments), adequate depth for variant detection was observed for a total of 99.7% (n = 152,798) of pathogenic variants (Table 1). The percentage of pathogenic variants with adequate depth for detection on WES was highest among Marfan/TAAD (99.8%) and lowest among XLID (98.5%) related gene panels. A total of 97.3% (n = 1491) of the pathogenic variants demonstrated adequate depth for detection across all 100 WES samples. The percentage of pathogenic variants with adequate depth for detection across all 100 WES samples was highest among PCD (98.2%) and lowest among XLID (73.9%) related genes. Of the diseases included in this analysis, genes involved in XLID represented the smallest number of pathogenic variants (n = 23). The lower proportion of pathogenic variants with adequate depth for detection for XLID may be a result of this small sample size as well as fewer analyzed alleles in that male subjects only have one copy of the X chromosome. The average percentage of bases covered ≥10X for the 100 WES samples was 94.8% (range 92.9–96.0), and the average depth per sample was 94X (range 80X-114X) (S2 Table). Furthermore, among these 100 WES samples, 98% bases were covered > 20X, 48% bases were covered > 100X and 0% of bases had no coverage.

**Table 1:** Exome sequencing coverage by disease type.

Disease	Pathogenic variant positions with ≥10X coverage across all 100 WES samples			Total pathogenic variant positions with ≥10X coverage in each WES sample		
	Positions covered, n	Positions assessed, n	% Positions covered	Positions covered, n	Positions assessed, n	% Positions covered
Cancer Susceptibility	1319	1350	97.7%	134,584	135,000	99.7%
Cardiovascular	51	53	96.2%	5280	5300	99.6%
Marfan/TAAD	50	52	96.2%	5187	5200	99.8%
PCD	54	55	98.2%	5481	5500	99.7%
XLID	17	23	73.9%	2266	2300	98.5%
All	1491	1533	97.3%	152,798	153,300	99.7%

TAAD, thoracic aortic aneurysms and dissections; PCD, primary ciliary dyskinesia; XLID, X-linked intellectual disability; WES, whole exome sequencing.

All pathogenic variants had at least some coverage on exome sequencing; however, there were 42 pathogenic variants (2.7%) that were covered <10X in at least one of the 100 WES samples. Subsequent review of these 42 pathogenic variants revealed that 11 alterations (26.2%) were in GC-rich regions (defined as GC-content >60%), 8 were in repetitive regions (19.0%) (defined as polymer stretching ≥9 basepairs), and 3 (7.1%) were located in regions with known pseudogene interference. For the remaining 20 pathogenic variants (47.6%), there was no obvious explanation for no/low exome coverage at the respective nucleotide position (Table 2). The pathogenic variant with the lowest level of coverage- c.325DELG (p.E109Kfs*3) in *PMS2*, a gene with high pseudogene homology- was detectable in 35 of the 100 WES samples. Low inter-sample variability was observed regarding the number of pathogenic variants not covered at ≥10X. Across all 100 exomes, the median number of variants lacking adequate coverage (<10X) in each sample was 5 (range 0–12). Coverage for all pathogenic variants per sample is provided in S3 Table.

**Table 2:** Pathogenic variant positions not covered across all 100 WES samples.

Gene	Nucleotide variant	Protein variant	Coding exon/intron	Exon %GC content	Intron %GC content^a^	Proposed reason for incomplete coverage	Samples covered ≥10X, n
*APC*	c.423-3T>A	NA	Intron 3	NA	28.0%	polymer stretch of 13 As	99
*ATM*	c.1236-2A>T	NA	Intron 8	NA	28.0%	polymer stretch of 15 Ts	77
*ATM*	c.1236-3_1236-2DEL	NA	Intron 8	NA	28.0%	polymer stretch of 15 Ts	77
*ATM*	c.2250G>A	p.K750K	CDS 13	42.1%	NA	NA	98
*ATM*	c.3994-2A>G	NA	Intron 25	NA	29.0%	NA	97
*ATM*	c.4776+2T>C	NA	Intron 30	NA	30.0%	NA	99
*ATM*	c.7875_7876DELTGINSGC	p.D2625_A2626delinsEP	CDS 52	39.6%	NA	NA	99
*ATM*	c.7913G>A	p.W2638*	CDS 52	39.6%	NA	NA	93
*ATRX*	c.109C>T	p.R37*	CDS 2	40.7%	NA	NA	98
*BRCA2*	c.6944_6947DELTAAA	p.I2315Kfs*12	CDS 12	38.6%	NA	NA	84
*BRCA2*	c.7007G>A	p.R2336H	CDS 12	38.6%	NA	NA	91
*BRIP1*	c.2392C>T	p.R798*	CDS 16	44.2%	NA	NA	44
*BRIP1*	c.2400C>G	p.Y800*	CDS 16	44.2%	NA	NA	57
*CBS*	c.1105C>T	p.R369C	CDS 10	67.9%	NA	GC-rich	88
*CDKN2A*	c.335_337DUPGTC	p.R112dup	CDS 2	72.3%	NA	GC-rich	51
*CUL4B*	c.2493G>A	p.T831T	CDS 19	38.2%	NA	NA	97
*EMD*	c.153dupC	p.S52Qfs*9	CDS 2	59.0%	NA	NA	97
*FBN1*	c.7C>T	p.R3*	CDS 1	60.4%	NA	GC-rich	99
*MECP2*	c.1152_1155DEL	p.P385Cfs*23	CDS 3	61.4%	NA	GC-rich	91
*MECP2*	c.1208DEL	p.P403Lfs*6	CDS 3	61.4%	NA	GC-rich	89
*MECP2*	c.1213C>T	p.P405S	CDS 3	61.4%	NA	GC-rich	95
*MRE11A*	c.21-6_26DEL12	NA	Intron 1-CDS 2	32.3%	NA	NA	85
*MSH2*	c.942+2T>C	NA	Intron 5	NA	27.0%	polymer stretch of 27 As	96
*MSH2*	c.942+3A>T	NA	Intron 5	NA	26.0%	polymer stretch of 27 As	94
*MSH6*	c.3172+1G>A	NA	Intron 4	NA	41.0%	NA	99
*MSH6*	c.3978_3979INSA	p.N1327Kfs*14	CDS 9	40.0%	NA	NA	98
*MSH6*	c.3980_3983DUPATCA	p.L1330Vfs*12	CDS 9	40.0%	NA	NA	99
*MSH6*	c.3984_3987DUPGTCA	p.L1330Vfs*12	CDS 9	40.0%	NA	NA	99
*PALB2*	c.226DELA	p.I76Yfs*101	CDS 4	40.1%	NA	NA	98
*PMS2*	c.2404C>T	p.R802*	CDS 14	55.3%	NA	pseudogene	50
*PMS2*	c.2500_2501DELATINSG	p.M834Gfs*17	CDS 15	52.1%	NA	pseudogene	97
*PMS2*	c.325DELG	p.E109Kfs*3	CDS 4	46.6%	NA	pseudogene	35
*PTEN*	c.802-2A>T	NA	Intron 7	NA	24.0%	polymer stretch of 15 As	99
*RAD50*	c.2156DUPT	p.E723Gfs*5	CDS 13	45.8%		variant located at edge of polymer stretch of 9 As	99
*RAD50*	c.2165DUPA	p.E723Gfs*5	CDS 13	45.8%		polymer stretch of 9 As	98
*RAD50*	c.2202DELC	p.M735*	CDS 13	45.8%		NA	98
*RET*	c.2410G>A	p.V804M	CDS 14	64.7%		GC-rich	98
*RPGR*	c.28+1G>A	NA	Intron 1	NA	75.0%	GC-rich	81
*SLC16A2*	c.453DELC	p.E152Sfs*6	CDS 1	68.4%		GC-rich	97
*STK11*	c.608DUPC	p.F204Vfs*62	CDS 5	68.6%		GC-rich	86
*STK11*	c.913C>T	p.Q305*	CDS 7	60.3%		GC-rich	99
*TTN*	c.59933-1G>C	NA	Intron 267	NA	39.0%	NA	83

NA, not available.

^a^ Intron % GC content for a 100 basepair window surrounding the variant

The lengthiest alterations assessed in this dataset were a 40-nucleotide deletion in *BRCA1*, and a 20-nucleotide duplication in *BARD1*. Since this study was based on coverage analysis at respective nucleotide positions and did not directly assess the performance of the exome hybridization or the alignment and variant calling algorithms to detect these deletions and duplications, it is difficult to know whether these would truly have been detected. However, based on a retrospective analysis of 500 WES cases performed at Ambry Genetics indels larger than 40 nucleotides accounted for 2.6% of positive results [6], demonstrating that such alterations are detectable by WES at our laboratory.

To validate study findings, a similar analysis was performed against coverage data from 60,706 exomes available through the Exome Aggregation Consortium (ExAC) [15]. The ExAC database was queried for the percentage of samples with coverage ≥10X at the first nucleotide position for each pathogenic variant. Considering that coverage was assessed among 60,706 individual ExAC WES samples for each alteration (93,062,298 individual assessments), a total of 98.6% (n = 91,743,296) of pathogenic variants demonstrated adequate depth for detection (S4 Table). A total of 86.2% (n = 1321) of the pathogenic variants demonstrated adequate depth for detection in ≥99% (n = 60,099) samples. Twenty-five percent (388/1533) of pathogenic variants in this analysis were reported in ExAC, demonstrating the ability of WES to detect at least a portion of these alterations in actuality.

To further compare the analytic sensitivity of targeted panels to WES, our internal database was queried for patients who underwent previous targeted panel testing, either through Ambry Genetics or an outside laboratory, prior to WES at Ambry. Sixteen patients had a total of 21 alterations detected on targeted panel testing, all of which were detected on WES at Ambry (S5 Table). Though limited in size, this dataset also demonstrates the ability of WES to equally detect alterations reported on targeted panel testing.

Recently, Park *et al*. investigated the performance of exome sequencing for the 56 genes in the American College of Genetics and Genomics’ incidental finding recommendation by determining coverage at the nucleotide positions for all 18,336 nucleotide variants annotated in HGMD for these genes [16]. Authors identified inadequate coverage for the majority of variants in 7 genes (*SDHC*, *SDHD*, *GLA*, *TGFBR2*, *COL3A1*, *PMS2*, and *PCSK9*) and also identified six GC-rich exons with a high failure rate in their coverage analysis of 12 clinical exomes. In this study, adequate depth for detection was observed for 100% pathogenic variants in five of these genes: *SDHC* (n = 3), *SDHD* (n = 3), *GLA* (n = 1), *TGFBR2* (n = 4), and *COL3A1* (n = 3). *PMS2* is known to harbor several homologous exons; thus, WES is not expected to yield accurate results for this gene. While *SDHC* and *SDHD* also harbor homologous exons, alterations in these genes that were included in our dataset were not located in exons with significant homology. There were not any *PSCK9* alterations included in our dataset, as this gene is not analyzed as part of any targeted NGS panels at our laboratory. A limitation to the Park *et al*. data, as pointed out by the authors, is that only 7.5% of ‘disease causing’ variants in HGMD are actually pathogenic/likely pathogenic [17]. While our study reported on a lesser number of alterations, they are classified based on our clinical laboratory standards to be pathogenic or likely pathogenic [8]. The vast majority of benign variants are single basepair substitutions which are well represented in this data set, along with insertions and deletions. The set of pathogenic variants is likely to provide the most complete and heterogeneous representation of variant subtypes including increased representation of variants in critical domains which are most likely to impact function. While variant subtype (single basepair substitution, indel, etc.) is subject to different NGS detection rates, clinical classification of alterations is not expected to have any impact on their detectability. It should be noted that the exome enrichment technique as well as the bioinformatics aligning and variant calling pipeline may result in differing detection rates.

Reports from multiple groups, including ours, have shown that the diagnostic yield of exome sequencing varies by indication [6, 18]. In addition, studies comparing targeted NGS panels with exome sequencing are, for the most part, disease-specific. Studies have supported targeted NGS panel testing as a first-tier testing approach over exome sequencing for several diseases based on diagnostic yield, coverage, and cost-savings [19–22]. For example, Wang and colleagues published results from a study on the diagnostic yield of a clinically-validated targeted NGS panel testing for retinitis pigmentosa (RP) [23]. Based on the high diagnostic yield reported in their study (82%) and the distinct phenotype observed with RP, the authors propose targeted panel testing as the first-tier approach for RP and WES as a second-tier option in cases where a molecular diagnosis is not made via panel testing. However, this study did not include any cross comparisons with the diagnostic yield of exome sequencing for RP. Results from another recent study of the performance of NGS and WES for inherited eye disorders also supported the use of targeted panel testing over WES based on superior analytic sensitivity and high diagnostic yield of the panel [24].

For diseases with a lower diagnostic yield on targeted NGS panel testing and/or less distinct clinical phenotypes, the choice between panel and exome as the first-tier testing approach may not be as straightforward. The major advantages of exome sequencing over targeted NGS panel testing is the detection of alterations in newly characterized genes, potential for novel gene characterizations, and ability to sequence nearly all genes in the genome. The rate of newly discovered gene characterizations is increasing rapidly and OMIM phenotypes for which the molecular basis is known almost doubled in the recent 6 years [25]. For each disease represented in this study, there have been published reports of novel genes after the NGS panels became clinically available. For example, approximately 30 genes have been associated with PCD, and a number of these have recently been discovered by WES [26–34]. According to current estimates, characterized PCD genes account for 66% of PCD cases, leaving the remaining third unexplained. Based on results from our study, WES coverage depth was adequate for detection for close to all (99.7%) pathogenic variants identified on targeted NGS panel testing, along with newly-discovered PCD genes not yet available on targeted NGS panels and identified potentially novel genes. An additional advantage of exome sequencing is the option for data reanalysis at a later point in time. Furthermore, the diagnostic yield of exome sequencing can be further maximized with approaches such as trio sequencing [6] and augmented exome sequencing [35].

Some important limitations of this study should be noted. Even though variants were detected in actual patients undergoing multigene panel testing and compared with coverage data from actual clinical exomes, this analysis is still theoretical and limited by the fact that variants studied were not directly detected prospectively on WES. While variants are confirmed with an orthogonal method prior to reporting WES results, the well-known disadvantage of exome sequencing compared to panel testing is the possibility of false-negative results as it is not feasible to confirm all no/low coverage regions across the exome without significantly impacting cost and turn-around-time. The results herein quantify the risk for false negatives on exome versus panel testing. Due to variations between exome enrichment platforms and bioinformatics methods, another limitation to this data is that it is based on the performance of SeqCap EZ VCRome 2.0 enrichment platform in combination with Ambry Genetics’ custom bioinformatics pipeline, and therefore is not generalizable to other labs using the same platform, or to other WES platforms. In addition, several alterations in genes containing regions of high sequence homology were included in this data set such as *CHEK2*, *MYH7*, and *PMS2*. Therefore, it is possible that alterations in these regions would have escaped detection if the reported coverage at the respective nucleotide position was representative of a homologous region rather than the actual gene. One final limitation to this study is that copy number variations were not assessed. Future studies are needed to directly compare the analytic and clinical sensitivity, cost analysis, and counseling implications for panels vs. WES. Examples include a two-arm study where patients are randomized to a targeted NGS panel vs. WES approach or a study where both a targeted panel and exome sequencing are performed on each patient concurrently. An important consideration is that factors beyond differences in technology and clinical and analytic sensitivity come into play in deciding to pursue targeted testing vs WES. For example, in hereditary cancer diagnostics, it is often imperative to have genetic testing results within a short turn-around-time, as results may impact surgical decisions such as lumpectomy vs. mastectomy in the setting of breast cancer or partial vs. total colon resection in the setting of colon cancer and polyposis syndromes [36, 37]. In addition to time-sensitive medical management considerations, there are also implications on genetic counseling practices. For example, targeted panels are unlikely to result in secondary findings, whereas this is a possibility for WES. Such factors are beyond the scope of this paper, but are important in considering testing approach.

## Conclusions

Despite current estimates that 90–95% exome-wide coverage is achieved with WES, results from this position-specific comparative coverage analysis limited to disease-causing variants identified through NGS panels demonstrate that exome sequencing is expected to perform well (≥98.5%) for a range of inherited diseases. If validated in follow-up studies, these data will help guide clinicians in deciding which type of testing to pursue for their patients. These data suggest the use of exome sequencing may achieve similar diagnostic yield when compared to panel based tests and, if cost and turn-around-time are comparable or favorable, that WES may be an appropriate first-tier option to consider when clinically indicated. The high level coverage achieved by WES reported herein, coupled with high rate of newly characterized and novel gene findings on exome (30% collectively) [6] demonstrates a major benefit of WES compared to panel testing for Mendelian diseases.

## Supporting information

S1 Table Pathogenic variant coverage by gene. (XLSX)

S2 Table Coverage summary for 100 WES samples. (XLSX)

S3 Table Pathogenic variant position coverage for 100 WES samples. (XLSX)

S4 Table ExAC coverage comparison. (XLSX)

S5 Table WES samples with prior panel testing. (XLSX)
